# Whole-genome resequencing of *Cucurbita pepo* morphotypes to discover genomic variants associated with morphology and horticulturally valuable traits

**DOI:** 10.1038/s41438-019-0176-9

**Published:** 2019-08-11

**Authors:** Aliki Xanthopoulou, Javier Montero-Pau, Ifigeneia Mellidou, Christos Kissoudis, José Blanca, Belén Picó, Aphrodite Tsaballa, Eleni Tsaliki, Athanasios Dalakouras, Harry S. Paris, Maria Ganopoulou, Theodoros Moysiadis, Maslin Osathanunkul, Athanasios Tsaftaris, Panagiotis Madesis, Apostolos Kalivas, Ioannis Ganopoulos

**Affiliations:** 1 Department of Genetics and Plant Breeding, Aristotle University of Thessaloniki, Thessaloniki, 54124 Greece; 2 Department of Biochemistry and Molecular Biology, Universitat de València, 46022 Valencia, Spain; 3 Institute of Plant Breeding and Genetic Resources, Hellenic Agricultural Organization DEMETER (ex NAGREF), Thermi, Macedonia 57001 Greece; 4 Perrotis College, American Farm School, Thessaloniki, 57001 Greece; 5 Institute for the Conservation and Breeding of Agricultural Biodiversity (COMAV-UPV), Universitat Politècnica de València, Camino de Vera s/n, 46022 Valencia, Spain; 6 Department of Vegetable Crops and Plant Genetics, Agricultural Research Organization, Newe Ya’ar Research Center, Ramat Yishay, Israel; 7 Institute of Applied Biosciences (INAB), CERTH, Thermi-Thessaloniki, 57001 Greece; 8 Department of Biology, Faculty of Science Chiang Mai University, Chiang Mai, Thailand; 9 Center of Excellence in Bioresources for Agriculture, Industry and MedicineChiang Mai University, Chiang Mai, Thailand

**Keywords:** Structural variation, Natural variation in plants

## Abstract

*Cucurbita pepo* contains two cultivated subspecies, each of which encompasses four fruit-shape morphotypes (cultivar groups). The Pumpkin, Vegetable Marrow, Cocozelle, and Zucchini Groups are of subsp. *pepo* and the Acorn, Crookneck, Scallop, and Straightneck Groups are of subsp. *ovifera*. Recently, a de novo assembly of the *C. pepo* subsp*. pepo* Zucchini genome was published, providing insights into its evolution. To expand our knowledge of evolutionary processes within *C. pepo* and to identify variants associated with particular morphotypes, we performed whole-genome resequencing of seven of these eight *C. pepo* morphotypes. We report for the first time whole-genome resequencing of the four subsp. *pepo* (Pumpkin, Vegetable Marrow, Cocozelle, green Zucchini, and yellow Zucchini) morphotypes and three of the subsp. *ovifera* (Acorn, Crookneck, and Scallop) morphotypes. A high-depth resequencing approach was followed, using the BGISEQ-500 platform that enables the identification of rare variants, with an average of 33.5X. Approximately 94.5% of the clean reads were mapped against the reference Zucchini genome. In total, 3,823,977 high confidence single-nucleotide polymorphisms (SNPs) were identified. Within each accession, SNPs varied from 636,918 in green Zucchini to 2,656,513 in Crookneck, and were distributed homogeneously along the chromosomes. Clear differences between subspecies *pepo* and *ovifera* in genetic variation and linkage disequilibrium are highlighted. In fact, comparison between subspecies *pepo* and *ovifera* indicated 5710 genes (22.5%) with Fst > 0.80 and 1059 genes (4.1%) with Fst = 1.00 as potential candidate genes that were fixed during the independent evolution and domestication of the two subspecies. Linkage disequilibrium was greater in subsp. *ovifera* than in subsp. *pepo*, perhaps reflective of the earlier differentiation of morphotypes within subsp. *ovifera*. Some morphotype-specific genes have been localized. Our results offer new clues that may provide an improved understanding of the underlying genomic regions involved in the independent evolution and domestication of the two subspecies. Comparisons among SNPs unique to particular subspecies or morphotypes may provide candidate genes responsible for traits of high economic importance.

## Introduction

The gourd family, *Cucurbitaceae*, includes a number of economically important vegetable crops, of which five have a worldwide distribution and importance^1^. These are cucumber (*Cucumis sativus* L.), melon (*Cucumis melo* L.), watermelon (*Citrullus lanatus* (Thunb. Matsum. & Nakai), and three species of pumpkins and squash (*Cucurbita pepo* L., *Cucurbita moschata* Duchesne, and *Cucurbita maxima* Duchesne). Although the fruits of all five of these cucurbit crops are highly diverse, pumpkins and squash are extraordinarily so, varying widely in size, shape, surface topography, color, and color pattern^2^.

The dozen or so species of *Cucurbita* are diploids with 20 pairs of chromosomes (2*n* = 2*x* = 40)^3^. *Cucurbita pepo* is phenotypically the most polymorphic species of the genus^4^, containing eight edible-fruited groups of cultivars (Groups), also known as fruit-shape morphotypes, with fruit shape being a polygenically inherited trait that changes little during fruit growth and is of utmost consumer importance^5^. Based on molecular genetic polymorphisms^4^, these eight Groups are distributed equally into two subspecies, *C. pepo* subsp. *pepo* and *C. pepo* subsp. *ovifera* (L.) Decker (=subsp. *texana* (Scheele) Filov). Subspecies *pepo* contains the Cocozelle, Pumpkin, Vegetable Marrow, and Zucchini Groups, while subsp. *ovifera* contains the Acorn, Crookneck, Scallop, and Straightneck Groups^4^.

*Cucurbita pepo* subsp. *pepo* has not been discovered in the wild, but is thought to have originated in Mexico and encompasses most of the cultivated germplasm. *Cucurbita pepo* subsp. *ovifera* grows wild in the southeastern and central United States, and encompasses much of the remaining cultivated germplasm. The two subspecies were domesticated independently^4^. A third subspecies, *C. pepo* subsp. *fraterna*^6^, is not cultivated and grows wild in northeastern Mexico. Subsp. *fraterna* is considered by some to be the wild progenitor of subsp. *pepo*^7^.

The flowers, fruits, and seeds are larger in subsp. *pepo* than in subsp. *ovifera*^8^. The two subspecies also differ markedly in fruit surface topography, often being ribbed in subsp. *pepo* and lobed, furrowed, and/or warted in subsp. *ovifera*. They both range widely in plant growth habit, sexuality, and parthenocarpic tendency. Three of the four morphotypes of subsp. *pepo*, Vegetable marrow, Cocozelle, and Zucchini, are of European and therefore post-Columbian origin, while the Acorn, Scallop, and Crookneck morphotypes of subsp. *ovifera* were established prior to the European contact with North America. Therefore, these subsp. *ovifera* morphotypes are likely to be more distinct from one another and more homozygous than their counterparts of European ancestry^6^. Fruits of the Pumpkin Group (subsp. *pepo*) are generally round and grown for their mature fruits for eating as well as for decoration, seed consumption, and extraction of seed oil. The fruits of the Acorn Group (subsp. *ovifera*) maintain the ancestral 1:1 length-to-width ratio, too, but are usually turbinate with ridges and furrows, and are consumed only when mature. Subsp. *pepo* is the more widely cultivated of the two subspecies, with its immature fruits being a popular vegetable around the world, especially those of the Zucchini Group. Not only Zucchini but also Cocozelle and Vegetable Marrow fruits are consumed when immature. These three Groups display elongated fruit shape, a trait that was selected in Southern Europe immediately after the arrival of various American pumpkins during the Columbian exchange in the early Renaissance. On the other hand, in subsp. *ovifera*, the elongated shape of the Straighneck and Crookneck Groups and the flattened shape of the Scallop Group were selected in North America long prior to 1492.

A multitude of genomic tools are available for the three other widely grown cucurbit crops, cucumber, watermelon, and melon. As a result, they have extensive genomic resources, including many molecular markers, sequenced genomes, and genotyping by sequencing (GBS) combined with genome-wide association studies (GWAS) that has led to the discovery of SNPs controlling horticulturally important traits^9–11^. Such tools have only recently been developed and implemented for pumpkin and squash, *Cucurbita*, and are beginning to accelerate gene discovery and breeding. For *C. pepo* especially, the potential is enormous, as simple sequence repeat (SSR) markers have indicated that this species contains by far the greatest genetic variation of the species of *Cucurbita*^10,11^.

*Cucurbita pepo* genomic resources were recently improved, initially with the generation of a single-nucleotide polymorphism- (SNP-) based genetic map leading to the discovery of quantitative trait loci (QTLs) related to vegetative and reproductive traits^12,13^. More recently, a high-density SNP-based genetic map has been developed by GBS using a RIL (recombinant inbred line) population from the inter-subspecific cross Zucchini × Scallop (subsp. *pepo* × subsp. *ovifera*^14^). Transcriptome sequencing efforts and mutant collections provide both genomic resources and insights into the regulation of fruit morphological and quality traits^15–17^. Recently, too, a de novo assembly of the *C. pepo* genome, a high coverage transcriptome of *C. pepo* and 40 transcriptomes of 12 species of *Cucurbita* have been published with comparative and phylogenetic analyses, indicating that *Cucurbita* originated from a whole-genome duplication event^18^. The assembly of the genome was conducted from the genomic DNA of a *Cucurbita pepo* subsp. *pepo* morphotype green Zucchini Spanish variety (accession BGV004370/MU‐CU‐16 from the COMAV-UPV Genbank). The assembly covers 93% of the estimated genome size, it is organized in 20 pseudochromosomes, has a scaffold N50 of 1.8 Mb, and includes 92.1% of a plant-specific database of 1440 conserved genes. Gene annotation was based on a multitissue transcriptome obtained from two cultivars: a *C. pepo* subsp. *pepo* morphotype green Zucchini MU-CU-16 and a *C. pepo* subsp. *ovifera* morphotype Scallop (accession BGV005382/V-CU‐196 from the COMAV-UPV Genbank)^18^.

Herein, we report for the first time whole-genome resequencing of seven of the eight edible-fruited morphotypes of *C. pepo*, the four belonging to subspecies *pepo* (Pumpkin, Vegetable Marrow, Cocozelle, and Zucchini) and three belonging to subspecies *ovifera* (Acorn, Crookneck, and Scallop). As separate domestication events occurred within the two subspecies, comparing the respective edible-fruited morphotypes of the two subspecies should result in a more comprehensive evaluation of genome variation within *C. pepo* and identification of genomic regions that have undergone differential selective pressures. These genomic resources could shed more light on evolutionary processes within this extremely polymorphic species and identify the genomic variations underlying economically important horticultural and morphological traits.

## Materials and methods

### Plant material

**Fig. 1:**
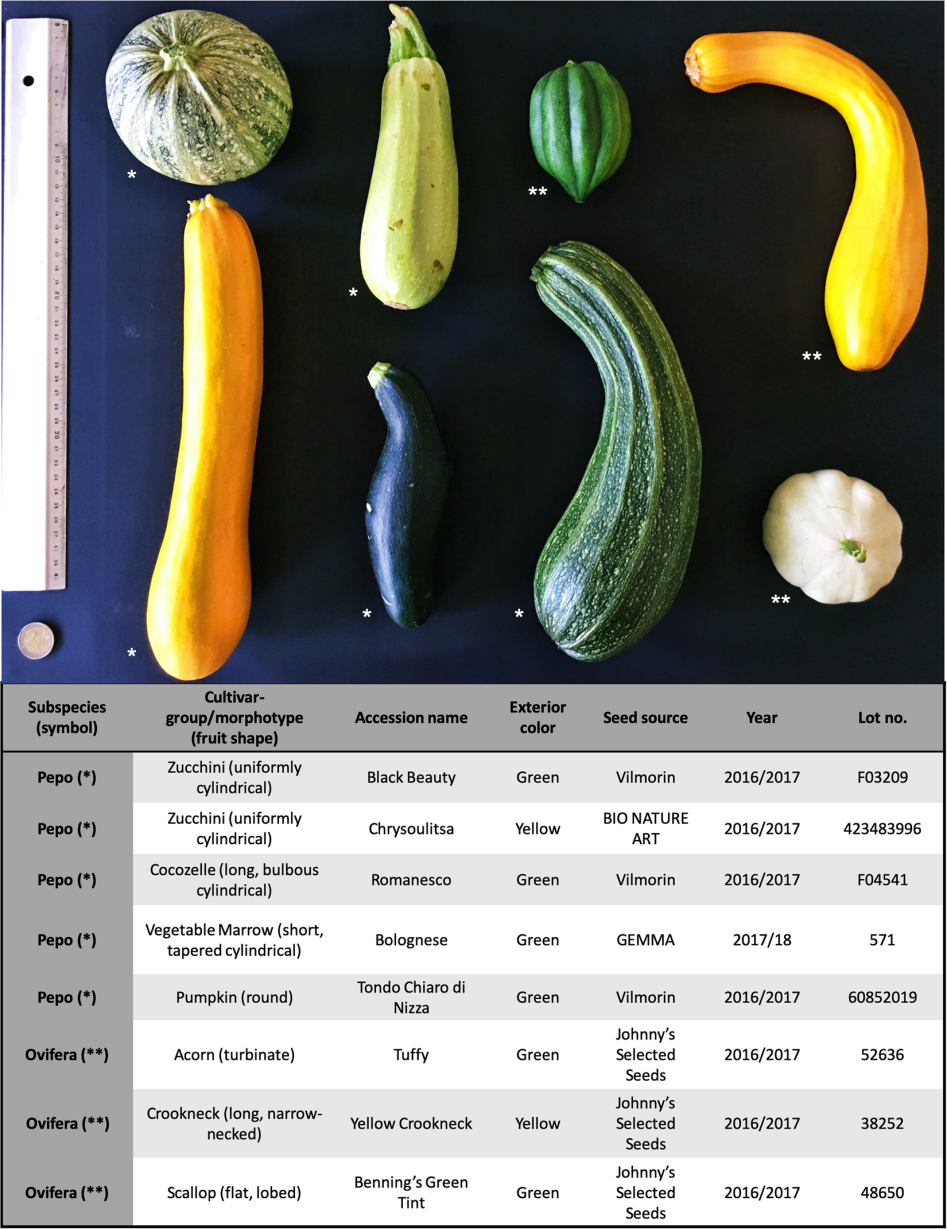
A fruit from each of the eight accessions of *Cucurbita pepo* used in this study. Top, left to right: “Tondo Chiaro di Nizza” (Pumpkin Group), “Bolognese” (Vegetable Marrow Group), “Tuffy” (Acorn Group), “Yellow Crookneck” (Crookneck Group). Bottom, left to right: “Chrysoulitsa” (Zucchini Group), “Black Beauty” (Zucchini Group), “Romanesco” (Cocozelle Group), and “Benning’s Green Tint” (Scallop Group). The accession information table is in the lower part, and subspecies *pepo* and *ovifera* are indicted by one (*) or two asterisks (**), respectively

Eight cultivars of *C. pepo* were used in this study (Fig. 1), five from the subsp. *pepo* and three from the subsp. *ovifera*, including at least one representative of seven of the eight edible-fruited cultivar groups. The subsp. *pepo* cultivars were “Romanesco” (Cocozelle Group), “Black Beauty” (Zucchini Group, with green fruits), “Tondo Chiaro di Nizza” (Pumpkin Group), “Bolognese” (Vegetable Marrow Group), and “Chrysoulitsa” (Zucchini Group, with yellow fruits). The subsp. *ovifera* cultivars were “Tuffy” (Acorn Group), “Yellow Crookneck” (Crookneck Group), and “Benning’s Green Tint” (Scallop Group) (see Fig. 1 for additional accession information). To integrate putative intra-cultivar variation, we used five plants from each cultivar. Seeds from each cultivar were planted in trays and plants were grown at the Experiment Stations of the Plant Breeding and Genetic Resources Institute (Hellenic Agriculture Organization (HAO), Demeter, Greece) under the same standard field conditions as previously described^17^. For library construction, young leaf samples were used for DNA extraction^19^, pooling leaves of the five plants per cultivar. For the detection of putative intra-cultivar variation we used a set of six inter SSR primers as described previously by Xanthopoulou et al^20^. PCR reactions and gel electrophoresis were conducted according to the authors above.

### Library construction and whole-genome resequencing

The extracted DNA was fragmented with a Bioruptor instrument (Thermo Fisher Scientific, Waltham, MA, USA) to generate 200–300 bp fragments. Libraries were then prepared as follows: first, the DNA fragments were subjected to end-repair and A-tailing; second, the resulting DNA was ligated with bubble adapters that contained a barcode sequence, and then amplified with PCR. Quality control was carried out with Agilent 2100 Bioanalyzer System (Agilent Technologies, Santa Clara, CA, USA) to assess the fragment size and with a Qubit dsDNA HS Assay Kit (Thermo Fisher Scientific) to measure the DNA library concentrations. Qualified libraries were pooled together to form single-stranded DNA circles and then DNA nanoballs were generated with rolling circle replication. The final DNA nanoballs were loaded onto a sequencing chip and were sequenced with the BGISEQ-500 platform (BGI-Tianjin). The BGISEQ-500 is a short-read sequencing platform, developed by BGI (Beijing Genomics Institute), that works by combinatorial probe-anchor synthesis that combines DNA-Nanoball arrays with stepwise sequencing using DNA polymerase on a flow cell. Comparative analysis between HiSeq2500 and BGISEQ-500 platforms have indicated overall comparative accuracy and sensitivity, and superior sensitivity for the BGISEQ-500 platform for SNP detection in DNA samples^21,22^.

### Reads pre-processing, read mapping, and SNP calling

From the pair-end 150 bp sequence data generated from the BGI platform, low-quality reads, adapter contamination, and duplicated reads and short reads (length < 35 bp) were removed. First, the adapter sequence in the raw data was removed, and low-quality reads that had too many Ns or a low base quality were discarded. This step produced the “clean data.” Second, the Burrows–Wheeler Aligner (BWA)-MEM algorithm in the BWA software (https://sourceforge.net/projects/bio-bwa/files/)^23^ was used to perform the alignment. The clean sequencing reads were aligned to the reference *C. pepo* genome v.4.1 (ftp://cucurbitgenomics.org/pub/cucurbit/genome/Cucurbita_pepo/)^18^. The binary alignment/map files were required for certain processes, such as fixing mate information of the alignment, adding read group information, and removing duplicate reads caused by PCR. SNP calling was performed using the SOAPsnp v.1.03 (http://soap.genomics.org.cn/soapsnp.html)^24^, SAMtools v.0.1.5-22 (http://www.htslib.org/download/)^25^, and GATK v.3.2 (https://www.broadinstitute.org/gatk/)^26^. Following this, filters were applied to obtain more confident variant results. Subsequently, AnnoDB software by the Bejing Genomics Institute was used to annotate the confident variant results. Additionally, genotypes with a depth lower than 10 reads or quality lower than 20 were set to missing, and non-biallelic variants or those with more than 30% of missing data were filtered out for subsequent analysis.

### Genomic variation

Genetic diversity (*π*), linkage disequilibrium (*r*^2^), and number of SNPs and proportion of heterozygous and homozygous genotypes were calculated for the eight accessions, as well as for each subspecies separately (*C. pepo* subsp. *pepo* and subsp. *ovifera*). TASSEL v.5^27^ was used to compute genetic diversity and Plink v.1.9^28^ for linkage disequilibrium. Pairwise *r*^2^ was obtained for all markers within 0.5 Mb and data were fitted using a local polynomial regression fitting implemented in R v. 3.3.3^29^. Background linkage disequilibrium (BLD) was estimated by bootstrap; 1000 replications were performed, and on each replication, *r*^2^ was calculated among 1000 randomly selected SNPs. BLD value was chosen as the upper interval of the 95% confidence interval of the *r*^2^ distribution. The number of SNPs and proportion of heterozygous and homozygous genotypes were calculated for each accession by using a custom Python library (https://github.com/JoseBlanca/variation/). Notice that each sequenced accession is the result of pooling five different plants. All genetic parameters were obtained for the whole genome and by using sliding windows along the genome. Circos v.0.69^30^ was used to create a genomic representation of the genetic indexes.

Genetic pairwise differentiation among accessions was obtained by applying Kosman and Leonard’s genetic distance^31^ using our custom Python library. Kosman and Leonard’s method is suitable for co-dominant markers in diploid individuals. An unrooted network based on the distance matrix was built using SplitsTree 4^32^ using the Neighbor-net algorithm. Genetic distance between subspecies was estimated by using Weir and Cockerham’s Fst using vcftools v.0.1.15^33^.

### Genetic variation on candidate genes

To explore the genetic variability across the eight accessions, and to study the potential effect of the genetic changes, each SNP along the genome was annotated based on its predicted effect on the gene using SNPeff v.4.3^34^. Several candidate genes underlying QTLs controlling horticultural traits of interest have been described in *C. pepo*^14,32^. Based on these studies, a total of 37 candidate genes related to flowering, fruit and flesh color, and leaf morphology were selected. Also, 18 key candidate genes related to fruit morphology, as has been shown in other fruit species, that is, tomato or melon, were also included in this study in order to explore polymorphism with potential implications for the genetic differences among accessions.

Recently, a group of proteins carrying a TONNEAU1 recruiting motif (TRM) has been shown to interact with OVATE to regulate cell division patterns during ovary development that alter final fruit shape^35^. In order to identify the proteins carrying TRM motifs in *C. pepo*, we performed a protein BLAST search (*blastp*) against all described TRM proteins in *Arabidopsis thaliana*^36^ and *Solanum*^35^. Only *C. pepo* proteins with an *E*-value lower than 10^−50^ were considered for further analysis. A multiple alignment of all protein sequences from all three species was performed using Clustal Omega v.1.2.4^37^, and phylogenetic tree was built using IQ-TREE v.1.5.2^38^. We used ModelFinder to choose the best-fitting evolutionary model^39^. Tree support was assessed through the Shimodaira–Hasegawa approximate likelihood ratio test, a Bayes support and 1000 bootstrap support by using the ultrafast bootstrap (UFBoot)^40^. The search of conserved motifs was done using MEME Suite v.5.0.3^41^.

Additionally, in order to identify candidate genes that can undergo different selective pressures during the domestication processes that led to the establishment of subspecies *pepo* and *ovifera*, genetic differentiation (Fst) between these two subspecies, and genetic diversity and Tajima’s D within each subspecies were calculated for each gene along the genome using vcftools v.0.1.15^33^.

## Results and discussion

### Genome sequencing and polymorphism analysis

Eight accessions belonging to the seven of the cultivar groups (morphotypes) of *C. pepo* ssp. *pepo* (Pumpkin, Vegetable Marrow, Cocozelle, yellow Zucchini, and green Zucchini) and ssp. *ovifera* (Acorn, Scallop, and Crookneck) were sequenced (Fig. 1). Eight DNA libraries were constructed and approximately 76.37 Gb were generated. From 84 to 107 million of clean reads were obtained per accession (Table 1).,

**Table 1:** Quantity and distribution of reads and SNPs for each morphotype

Morphotype	No. of clean reads	Coverage	% Mapped	Variable sites			% Mis	Ho
				No.	% Biallelic	% Monoallelic		
Pumpkin	107,814,560	37.8	94.1	1,077,816	28.38	71.62	1.79	0.08
Marrow	84,887,546	29.8	93.9	1,136,856	39.13	60.87	6.01	0.12
Cocozelle	96,385,370	33.8	94.0	1,194,461	43.76	56.24	5.44	0.14
Zucchini (yellow)	101,593,207	35.7	94.7	1,045,520	75.02	24.98	2.91	0.21
Zucchini (green)	86,968,548	30.5	95.1	636,918	53.30	46.70	3.83	0.09
Subspecies *pepo*	95,529,846	33.5	94.4	1,018,314	47.92	52.08	4.00	0.13
Acorn	91,160,816	30.1	94.4	2,421,537	9.17	90.83	4.89	0.06
Scallop	93,992,800	32.9	94.9	2,620,106	27.61	72.39	5.34	0.20
Crookneck	106,734,784	37.4	95.0	2,656,513	22.60	77.40	2.84	0.16
Subspecies *ovifera*	97,296,133	33.5	94.8	2,566,052	19.79	80.21	4.36	0.14

Number of clean reads after cleaning, genome coverage obtained, percentage of mapped reads, number of variable sites compared to the reference genome (no. of variable sites), percentage of the variable sites that are biallelic (%, that is that have two alleles, one identical, and the other different from that of the reference genome) and monoallelic (%, that is that have only one allele, different to that of the reference genome), and percentage of missing data (Mis), and observed heterozygosity (Ho) for the 3,823,977 variable positions found along the genome for all the morphotypes. Average values for each subspecies are also shown. Notice that reference genome was developed from a *C. pepo* subps. *pepo* morphotype green Zucchini Spanish accession^18^

**Fig. 2:**
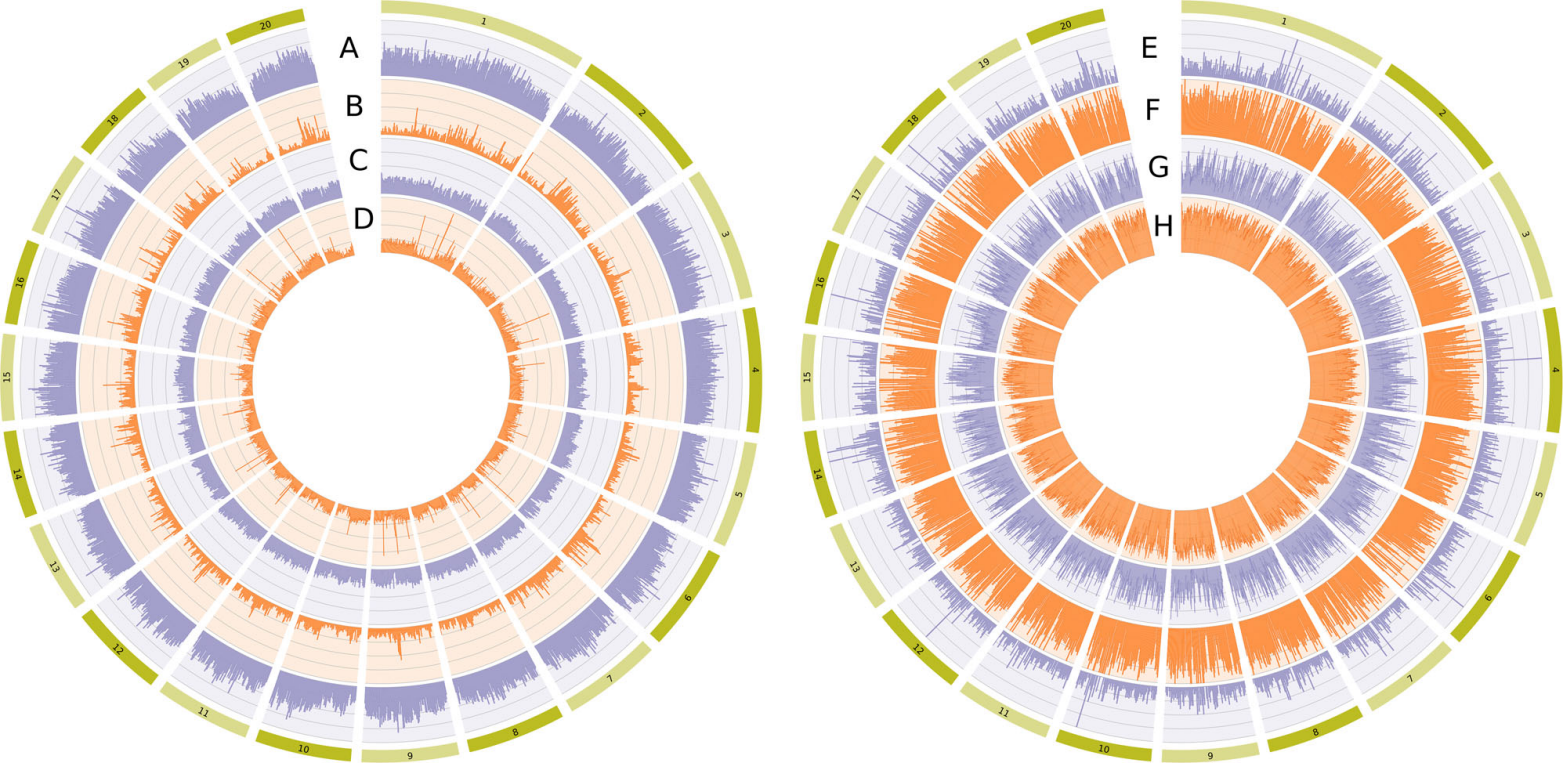
A circos diagram illustrating the genome-wide variations among 8 accessions. Circos plot showing the genomic distribution of **a** number of single-nucleotide polymorphisms (SNPs), **b** frequency of heterozygous SNPs, **c** frequency of homozygous SNPs for the allele not present in the reference genome, **d** number of genes, **e** number of SNPs with a high gene impact like stop codon gaining or frameshifts as predicted by SNPEff, **f** number of SNPs with a moderate gene impact like non-synonymous aminoacid changes, or non-disruptive frameshift, **g** linkage disequilibrium, and **h** genetic diversity. In all cases, 100 kb windows have been used, but for linkage disequilibrium (LD) a 50 kb window was used

We applied a high-depth resequencing approach that enables the identification of rare variants. The average depth of coverage varied from 29.8 to 37.8X depending on the accession, with an average of 33.5X. About 94.5% of the reads were mapped against the reference genome. Interestingly, despite the reference genome was derived from a *C. pepo* subsp. *pepo* morphotype Zucchini (green) Spanish accession, no differences in mapping percentage was found between subsp. *pepo* and *ovifera*. A total of 4,917,694 SNPs were found. After filtering out SNP genotype calls with low depth, non-biallelic SNPs and SNPs with more than 30% of missing data, 3,823,977 were kept for subsequent analysis. The proportion of small InDels was 19.72% (9.34 and 10.38% of insertions and deletions respectively). The number of SNPs per chromosome varied from 378,577 (Cp4.1LG01; Cp4.1 refers to the genome version 4.1 of *C. pepo*, and LG01 to the linkage group number 1) to 143,203 (Cp4.1LG19), and SNP density per kb ranged from 17.2 (Cp4.1LG02) to 19.8 (Cp4.1LG09) (Fig. 2).

Distribution of SNPs along the chromosomes was quite homogeneous (one-tailed *t* test *P* = 1.00 for all chromosomes), although a weak negative relationship was found between density of genes and density of SNPs (*R*^2^ *=* 0.02079, *P* < 0.001) as expected if the accumulation of SNPs is more likely in non-coding regions. According to their genomic positions (Table 2), 61% of SNPs were located in intergenic regions and 6% in exons, and the mean numbers of SNPs per kb were 16 and 9, respectively. Similar distribution of SNPs were observed among subspecies.

**Table 2:** Genomic location of SNPs

Location	No. of variants	%
Intergenic	2,370,305	60.97
Exon	247,852	6.38
Intron	1,031,861	26.54
Splice site region	26,534	0.68
5′-UTR	143,721	3.70
3′-UTR	67,431	1.73

*SNP* single-nucleotide polymorphism, *UTR* untranslated region

**Fig. 3:**
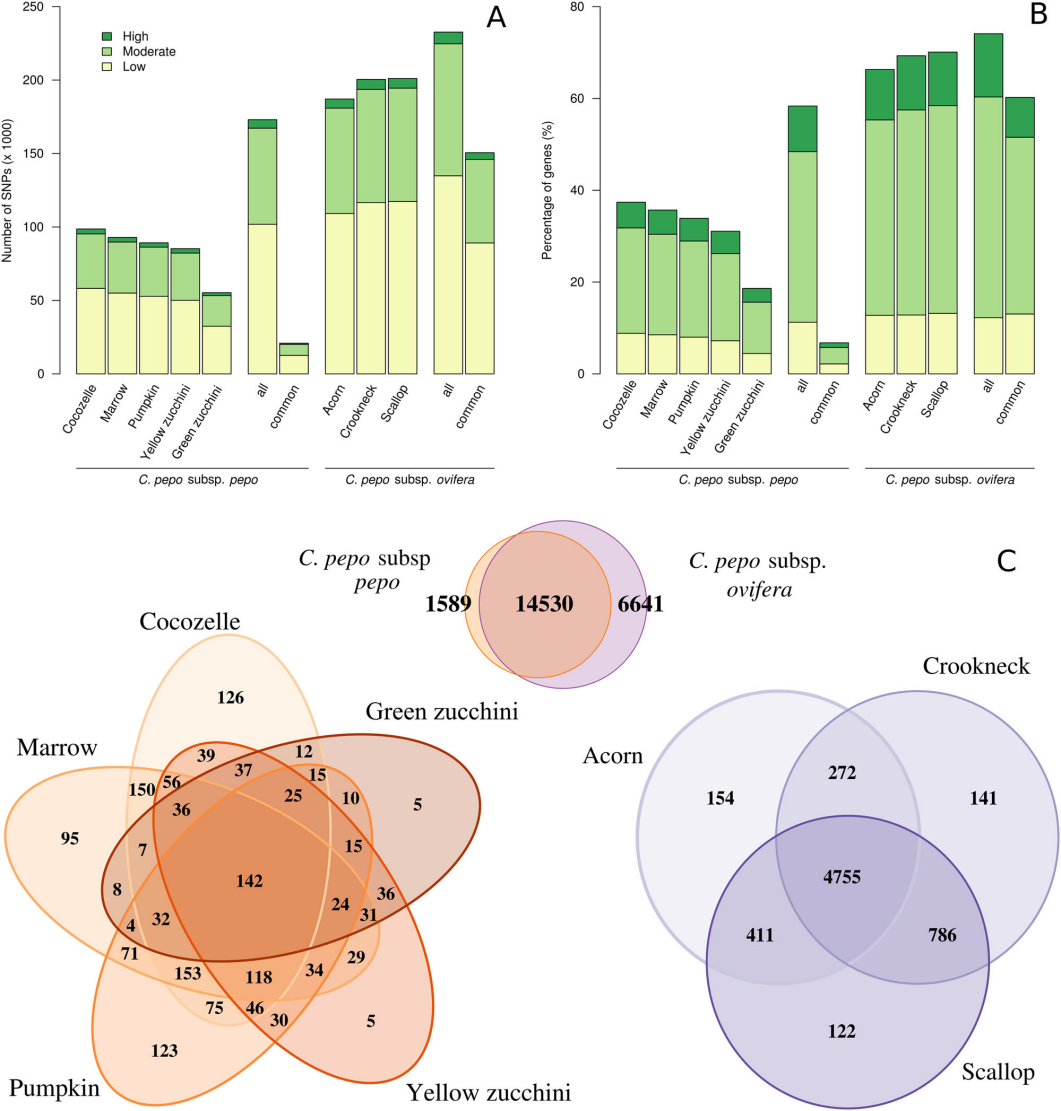
Single-nucleotide polymorphisms (SNPs) and percentage of genes with a SNP for each morphotype. Histograms of the number of SNPs obtained by comparison with reference genome (**a**) and percentage of genes with SNPs (**b**) for each morphotype classified according to their predicted effect. For each subspecies, the number of all and common SNPs/genes when considering all of the accessions. Venn diagrams (**c**) showing the number of genes with and SNPs with high or moderate effect when comparing both subspecies and when comparing morphotypes within subspecies

Based on the type of change and its predicted effect, it was found that the genomic ratio of non-synonymous/synonymous (Ka/Ks) changes was 0.78, whereas the transition/transversion ratio (Ts/Tv) was 1.63. The same values were obtained when considering each subspecies separately. Approximately 0.25% of the SNPs were predicted to have a high impact (e.g., stop codon gaining, frameshift), 2.79% a moderate (e.g., non-synonymous change, non-disruptive frameshift), and 4.19% a low impact (e.g., synonymous coding/start/stop, start gained). Figure 3 shows the number of SNPs (Fig. 3a) and the percentage of genes with a SNP (Fig. 3b) for each morphotype, classified according to their predicted effect. For each subspecies, the number of all and of common SNPs/genes when considering all morphotypes is also shown. Venn diagrams showing the number of genes with SNPs of high or of moderate effect when comparing both subspecies and when comparing morphotypes within subspecies are also included (Fig. 3c). These unique genes are listed in Supplementary File 1. As expected, the two accessions of the most recent and uniform morphotype, green and yellow Zucchini, which are also closer to the reference genome, which is derived from a green Zucchini, have a low number of genes with unique SNPs (five for the green Zucchini and five for the yellow Zucchini) with predicted effect (Fig. 3c). The green and yellow Zucchini accessions have 450 genes with differential SNPs with predicted effects that can be scrutinized to find significant mutations related to economically important traits (Fig. 3c). Furthermore, 36 genes with predicted-effect SNPs were common in the two Zucchini accessions and differ from the other *pepo* morphotypes, which could be associated with specific characteristics of this morphotype. Within subspecies *ovifera* (Fig. 3c), there appears to be morphotype-specific genes that possess SNPs with predicted effects that could be further studied and validated using a larger number of accessions of each morphotype, for identifying those that are indeed associated with morphotype-specific characteristics.

### Genome-wide genetic variation

**Fig. 4:**
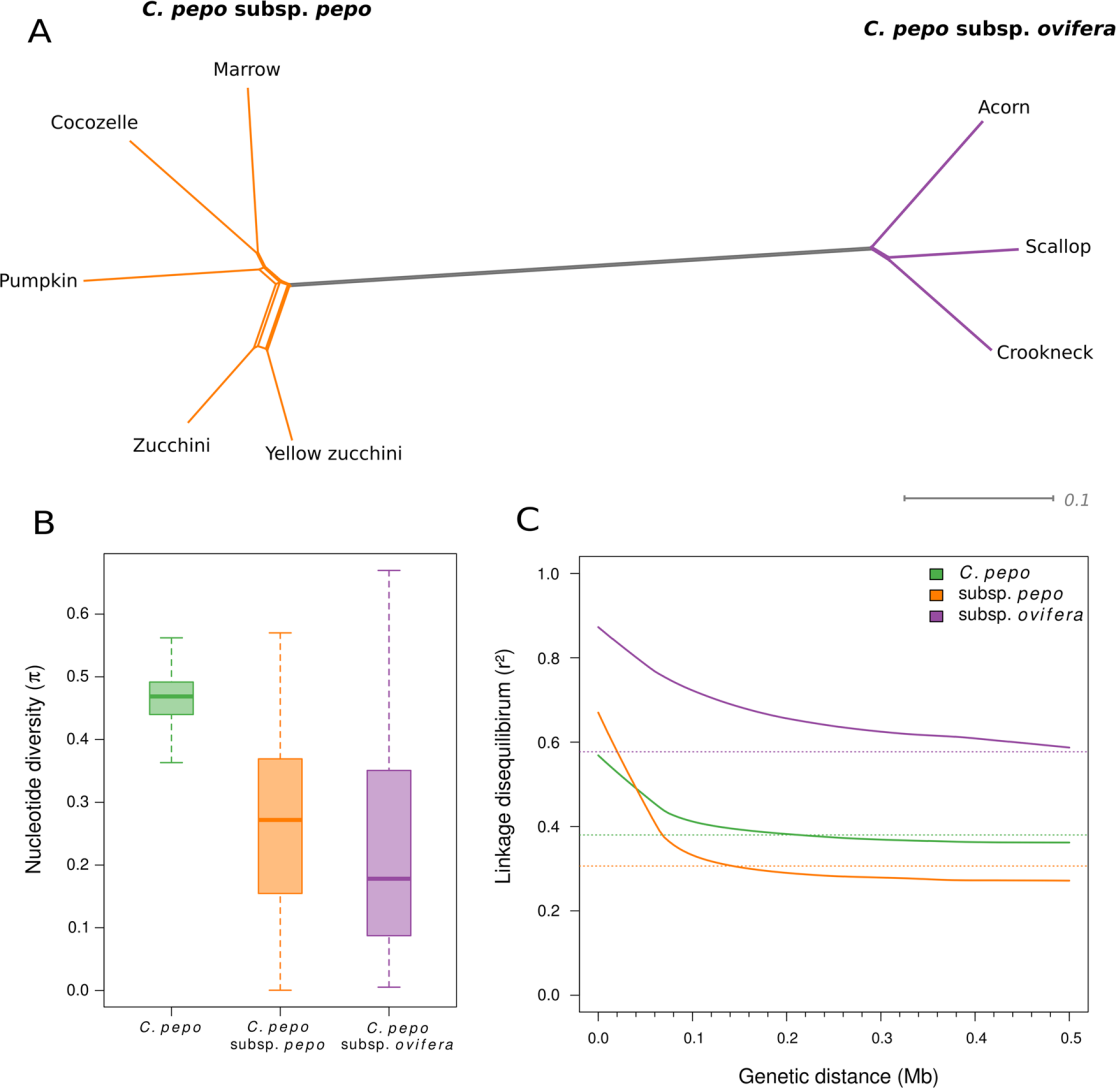
Genetic diversity and structure of eight accesions. **a** Phylogenetic network showing the relationship among the eight *Cucurbita pepo* accessions. **b** Boxplot of genetic diversity (*π*) of 1 kb windows and **c** linkage disequilibrium (*r*^2^) for the species, using all accessions, and for each subspecies. Horizontal dashed lines show the background linkage disequilibrium

**Fig. 5:**
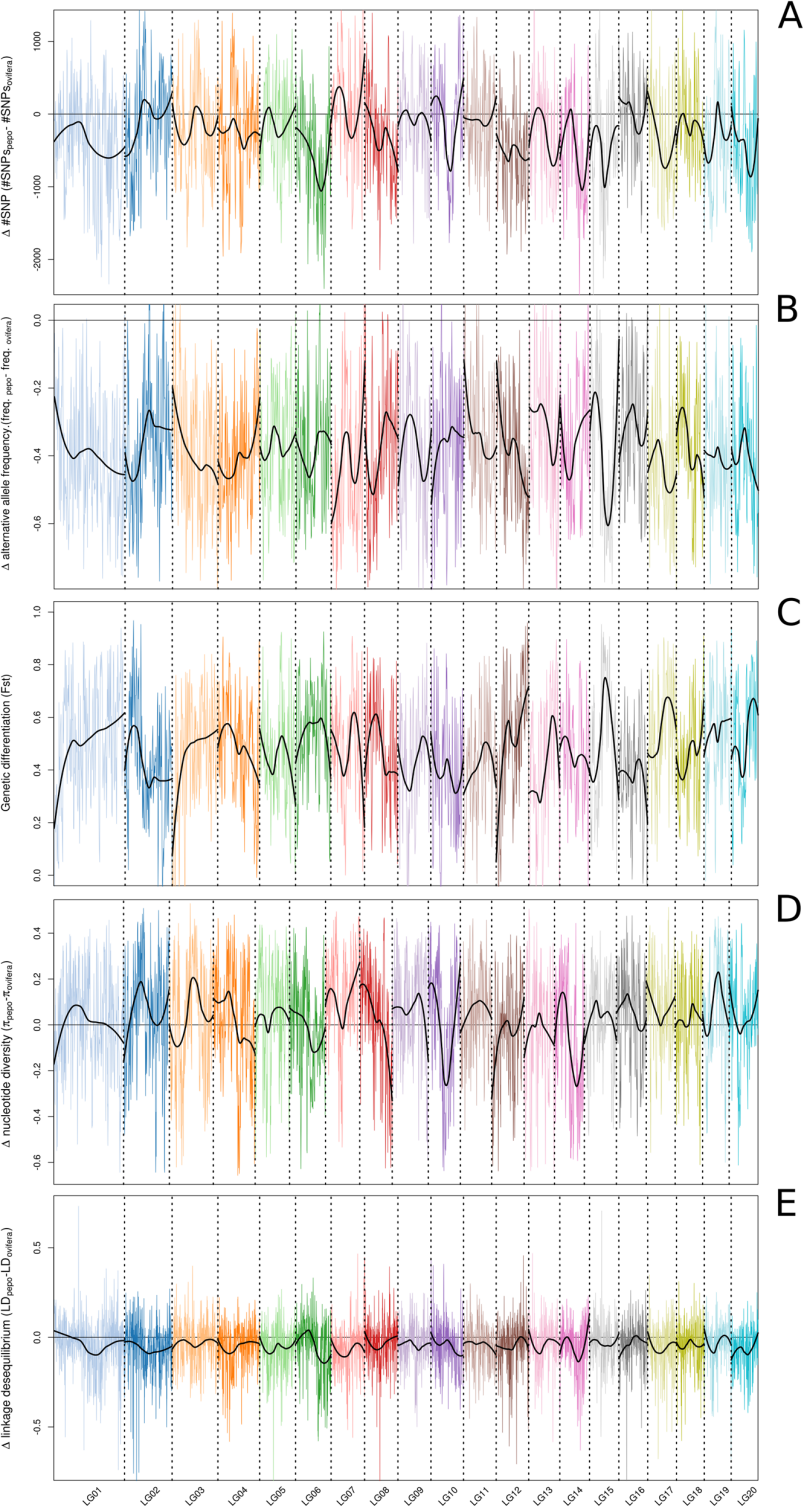
Genome wide differences between Cucurbita pepo subsp. pepo and subsp. ovifera. Differences in **a** number of SNPs, **b** frequency of the alternative allele (allele not present in the reference genome), **c** genetic differentiation, **d** nucleotide diversity, and **e** linkage disequilibrium between *Cucurbita pepo* subsp. *pepo* and subsp. *ovifera* along the chromosomes. Vertical dashed lines indicate the end/beginning of a chromosome. Solid horizontal line marks the absence of differences

The total number of SNPs within each accession varied from 636,918 in green Zucchini to 2,656,513 in Crookneck (Table 1). As expected, the three accessions belonging to the subsp. *ovifera* morphotypes (Acorn, Scallop, and Crookneck), which are more distant phylogenetically from the Zucchini reference genome (Fig. 4), presented almost twice as many SNPs as the morphotypes of subsp. *pepo* (>2M SNPs). Among the accessions of subsp. *pepo*, the one belonging to the Cocozelle Group had the highest number of SNPs. In contrast, the accessions belonging to the Zucchini Group had the lowest number of SNPs. The markedly high number of SNPs in *C. pepo* subsp. *ovifera* occurred throughout the whole genome, especially in large regions of chromosomes Cp4.1LG02, Cp4.1LG07, Cp4.1LG10, and Cp4.1LG16 (Fig. 5a). The phylogenetic network based on genetic distance among accessions showed little reticulation, indicating a low gene flow between accessions, but for the green and yellow Zucchini types (Fig. 4a). Average genetic diversity was similar between subspecies (Fig. 4b), but differences were found throughout the genome (Fig. 5d). Clear differences between subspecies were found in specific genomic regions, with higher genetic diversity in subspecies *pepo* (Cp4.1LG02, Cp4.1LG03, Cp4.1LG07, Cp4.1LG08, Cp4.1LG10, Cp4.1LG14, and Cp4.1LG19) or in subspecies *ovifera* (Cp4.1LG06, Cp4.1LG08, Cp4.1LG09, Cp4.1LG10, Cp4.1LG12, and Cp4.1LG14). Some of these underlying previously reported QTLs are involved in plant morphology and fruit color^14^. For example, genetic diversity was higher in ssp. *pepo* in the regions of QTLs involved in leaf and peduncle morphology (leaf incision, *Li_10* and immature peduncle length, *IPeLe_10*) and fruit rind and flesh color (immature fruit rind and flesh color, *ILRCo_10*, *IaRCo_10*, *IbFCo_10*, *IaFCo_10*, and mature fruit flesh color, *MaFCo_10*, Hunter parameters *L*, *a*, and *b*) in Cp4.1LG10, and involved in fruit rind and flesh color (mature rind and flesh color, *MbRCo_19* and MbFCo-19) in Cp4.1LG14 (previously referred to as LG19) (Fig. 5d).

**Fig. 6:**
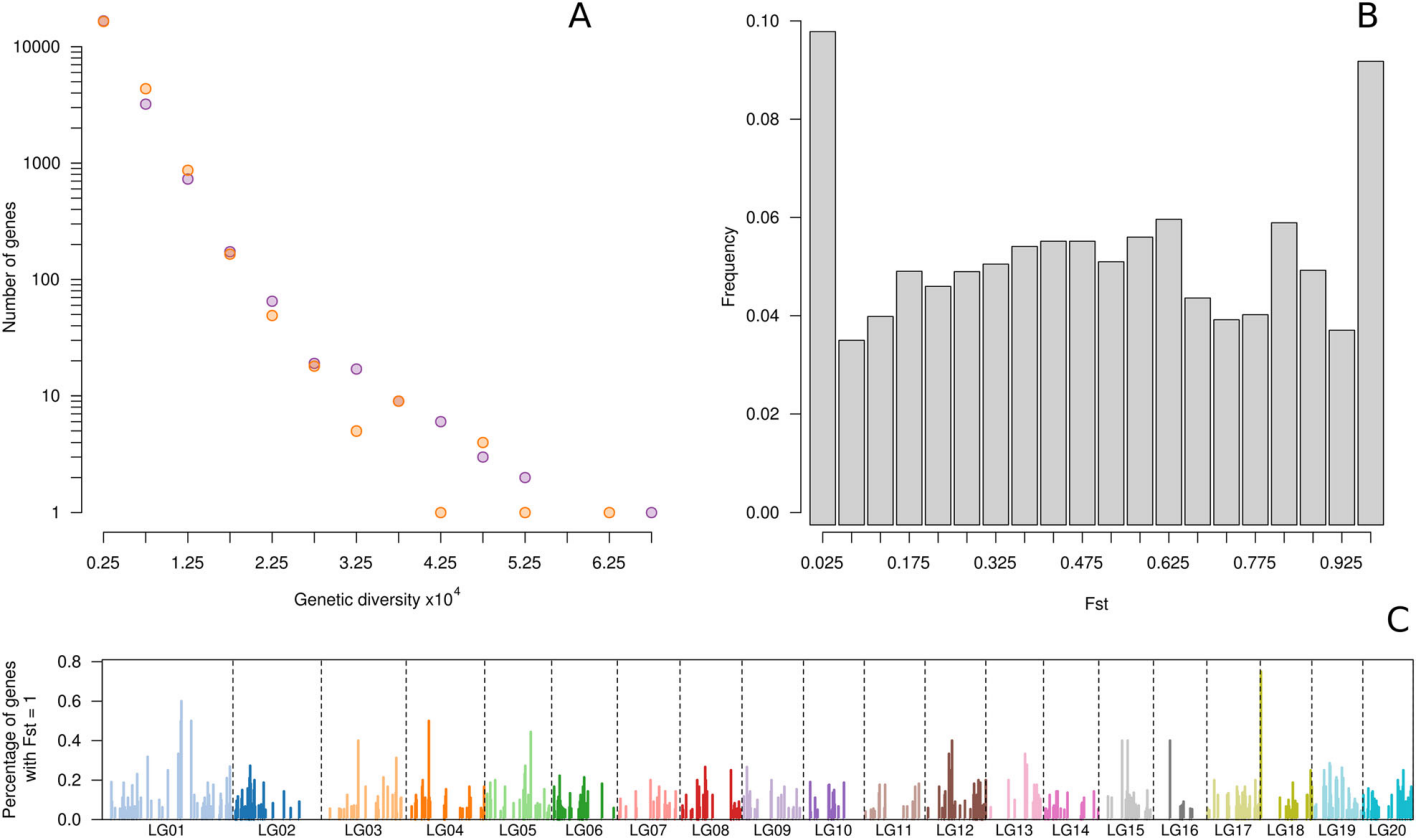
Genes and genetic diversity. **a** Number of genes with different levels of diversity in *Cucurbita pepo* subsp. *pepo* (orange) and subsp. *ovifera* (purple). **b** Histogram of genetic differentiation (Fst) by gene among subsp. *pepo* and subsp. *ovifera* and **c** distribution along the genome of genes with a Fst = 1

Gene diversity within *C. pepo* was also studied. Figure 6a indicates a similar number of genes with different levels of genetic diversity in each subspecies. Some specific genes were highly variable only in one subspecies (i.e., genes with a genetic diversity higher than the 99th percentile of genetic diversity of the subspecies) (Supplementary Files 2 and 3). There are specific gene groups more variable in subsp. *pepo* (119) or in subsp. *ovifera* (108) in all chromosomes, but these are more abundant in Cp4.1LG2, Cp4.1LG8, and Cp4.1LG11 (*pepo*) and Cp4.1LG3, Cp4.1LG12, and Cp4.1LG14 (*ovifera*). Some of these highly variable genes, in either species, are involved in abiotic and biotic stress responses, while others are involved in ion transport, in plant hormone metabolic pathways, and in plant/organ development and morphology. It should be mentioned that certain variable genes underlie previously reported QTLs^14^ involved in peduncle morphology (Cp4.1LG10g01340 located in Cp4.1LG10:3,351,368–3,352,316, within the interval of *IPeLe_10*), which is more variable in subsp. *pepo*, or involved in fruit shape (calcium-dependent lipid-binding domain, Cp4.1LG17g02010, located in Cp4.1LG17:1,396,894–1,398,462, within the interval of QTLs y *IFSh_12*, *IFLe_12*, *MFWi_12*, for immature fruit shape, for length, and for mature fruit width), which is more variable in subspecies *ovifera*. These genes have a high Tajima’s *D*, which suggests that they have been subjected to selection, so it is interesting to study them in detail to explain subspecies-specific differences.

### Linkage disequilibrium

LD was initially analyzed in the whole germplasm set. On a genome-wide average, LD was moderate (*r*^2^ < 0.6) even at very close distances (<1 kb), and decayed in this collection within 200 kb to a level below *r*^2^ = 0.4 (Fig. 4c). These values are higher than those reported for melon^11,42^. The previous studies with different melon collections show low LD (*r*^2^ < 0.5) within 0.5 or 1 kb^43,44^, and report a rapid LD decay within 100 kb to a level below *r*^2^ = 0.2^9–11^. Similar results were reported in watermelon^45^. The LD extent can vary according to different factors, including mating system, genetic drift, natural and artificial selection, recombination rate, population size and structure, and so on. Regarding the mating system, *C. pepo*, for example, has a high degree of cross-pollination by insects that it is also enhanced by its monecious sexual system. Melon is also considered an outcrossing species, and despite many current cultivars are andromonoecious, intercrossing have been suggested to occur during cultivar development by traditional farmers. Outcrossing lead to a decrease in LD, but other factors could explain the higher LD found in *C. pepo*, such as small population size due to the large plant size, genetic isolation between lineages, population subdivision, and so on, factors that contribute to the increase of LD. We have estimated the LD in a limited number of modern cultivars, likely subjected to inbreeding during the selection programs, which can also lead to increased LD. A higher number of accessions, representing not only modern cultivars but also old landraces, should be used to better estimate LD in *C. pepo*.

LD was also calculated separately for the two subspecies (Fig. 4c). In these cases, LD was more important in subspecies *ovifera* than in subspecies *pepo*, with *r*^2^ = 0.85 and *r*^2^ = 0.65, respectively, at very short distances (1 kb). Further, LD displayed a more rapid decay in subspecies *pepo*, within 140 kb to *r*^2^ < 0.3 and within 500 kb to *r*^2^ < 0.6 in subsp. *pepo* and *ovifera*, respectively. Differences in LD estimation in different germplasm collections have been reported previously. For example, in melon, differences in LD have been reported for the *inodorus* and *cantalupensis* horticultural groups, which represent different domestication and breeding histories^46^. Also in watermelon, lower LD was noted in African accessions as compared with those from the rest of the world, a consequence of a selective sweep or of a hitchhiking effect that has occurred in accessions developed out of the origin center^45^. Differences between *ovifera* and *pepo* accession could be a consequence of a higher divergence among the ovifera cultivars used and/or of the occurrence of a less strong selection in this group compared to that occurred in the more commercial subspecies *pepo*. These differences should be confirmed with a high number of accessions.

LD in subspecies *ovifera* was higher along the genome (Fig. 5e), but substantial variation in local LD patterns were observed among chromosomes. Interestingly, in chromosome Cp4.1LG06 and Cp4.1LG14, these preferential regions of high LD in *ovifera* are also associated with an increase in genetic diversity. Usually genetically more diverse regions show faster LD decay than that of less diverse regions. This high LD regions associated to high diverse regions could be the result of recent introgressions or of the occurrence of inversions.

### Genetic differentiation

The pairwise Fst statistic, associated with the genetic differentiation among groups, was calculated between subspecies (Figs. 5c and 6b). SNPs with high Fst were measured in some chromosomes. It is remarkable that a high density of genes with high Fst was found on chromosomes Cp4.1LG01, Cp4.1LG04, and Cp4.1LG18 (Fig. 6c). Comparison between subspecies *pepo* and *ovifera* displayed 5710 genes (22.5%) with an Fst > 0.80, and 1059 genes (4.1%), with an Fst = 1.00 distributed in all chromosomes (Supplementary File 4), revealing therefore good candidate genes for marking genomic regions that were fixed during the independent domestication of the subspecies, or had already been fixed in their respective wild ancestral populations long prior to domestication. Interestingly, some of these genes are located in the regions defined by QTLs involved in flesh and rind color (*IaFCo_10*, *IbFCo_10*, *IaRCo_10*, *ILRCo_10* Cp4.1LG10 4,515,143–5,185,624, and *MaFCo_19, MbFCo_19 MbRCo_19*, Cp4.1LG14 1,941,049–3,268,099), and in fruit shape (*MFWi_12*, *IFLe_12*, *IFSh_12*, *MFLe_12*, Cp4.1LG17 921,255–1,670,231) and ribbing (*IFRib_3*, immature fruit ribbing, Cp4.1LG03 8,457,590–8,877,700). In some of these regions, candidate genes were not previously reported so these differentiated genes could be the ones responsible for subspecies-specific differences in these traits. Also, some specific genes, previously reported as candidates of other QTLs, are differentiated between subspecies, such as the gene Cp4.1LG19g07200 (ethylene-responsive transcription factor 4-like) underlying the *DFeF_9* (days to female flowering), which is involved in early flowering. These co-locations of differentiating SNPs among these groups and QTLs that are involved in major traits could be valuable to identify responsible genes of major traits in *C. pepo*.

### Variation in genes of interest

We selected a set of 37 genes underlying QTLs previously described as involved in leaf morphology, flowering time, and fruit quality, and studied their variation among the various morphotypes of both subspecies. The entire list of genes with SNPs that have predicted effect are summarized in Supplementary File 5, while part of this list that contains genes of interest that have SNPs with HIGH predicted effect are given in Table 3.

**Table 3:** Genes of horticultural interest

Gene	Trait	GeneID	Genomic location	No. of variants in the gene with predited effect	No. of variants in the gene with HIGH effect	No. of variants in the gene with MODERATE effect	No. of variants upstream/downstream of the gene with predicted effect	
Cauliflower *OR* orange protein	Flesh color	Cp4.1LG13g00690	Cp4.1LG13:628,094–633,068	60	1	2	97	
Ethylene-insensitive 3 (*EIN3*) (1)	Flowering	Cp4.1LG04g11790	Cp4.1LG04:8,670,547–8,672,394	8	1	2	0	
Flowering time control protein (*FPA*)	Flowering	Cp4.1LG17g10910	Cp4.1LG17:8,241,363–8,252,364	339	4	86	201	
Squamosa promoter binding protein-like (2)	Flowering	Cp4.1LG17g10620	Cp4.1LG17:8,083,301–8,086,452	81	1	7	167	
Unusual floral organs	Flowering	Cp4.1LG20g02860	Cp4.1LG20:1,553,934–1,555,718	56	2	7	117	
Protein IQ-DOMAIN (2)	Fruit morphology	Cp4.1LG03g08410	Cp4.1LG03:2,815,265–2,818,054	38	1	0	40	
Tonneau recruiting motif	Fruit morphology	Cp4.1LG03g01040	Cp4.1LG03:1,235,448–1,237,683	40	1	10	76	
Tonneau recruiting motif	Fruit morphology	Cp4.1LG03g06270	Cp4.1LG03:4,147,734–4,152,353	69	4	6	193	
Tonneau recruiting motif	Fruit morphology	Cp4.1LG05g00950	Cp4.1LG05:499,361–506,004	71	1	9	94	*
Tonneau recruiting motif	Fruit morphology	Cp4.1LG08g10310	Cp4.1LG08:7,948,336–7,951,922	65	2	11	65	
Tonneau recruiting motif	Fruit morphology	Cp4.1LG10g04070	Cp4.1LG10:1,593,038–1,598,092	54	1	4	119	
Tonneau recruiting motif	Fruit morphology	Cp4.1LG12g10340	Cp4.1LG12:9,512,443–9,518,623	46	2	5	46	*
Tonneau recruiting motif	Fruit morphology	Cp4.1LG15g00200	Cp4.1LG15:105,059–109,560	60	2	10	23	*
Tonneau recruiting motif	Fruit morphology	Cp4.1LG15g01180	Cp4.1LG15:901,114–905,856	40	2	12	57	*
Tonneau recruiting motif	Fruit morphology	Cp4.1LG17g00460	Cp4.1LG17:325,022–334,267	93	1	9	103	

Selected genes, genomic locations, traits affected, number of changes predicted in total and with high and moderate effect, and number of changes 5 kb upstream or downstream of the genes that can have a potential effect are shown. For the TON1 recruiting motif proteins, asterisks identify those proteins that are phylogenetically close to TRM proteins of tomato known to interact with OVATE

Some of these genes have mutations with high impact in the 5′-, 3′-untranslated region, exons, and introns (Suppl. Files 6–25). Four of them are *ethylene-insensitive 3* (*EIN3*), the *flowering time control protein* (*FPA*), a *squamosa promoter binding protein-like*, and the *unusual floral organs* (*UFOs*), which have been previously reported to be involved in flowering^14^. All the genotypes of both subspecies were heterozygous for the four mutations (frameshift variant) with high impact in *FPA* (Cp4.1LG17g10910) (Supplementary File 6). The high impact mutation in a splice donor site in the squamosa promoter binding protein-like (Cp4.1LG17g10620) was more frequent in the *ovifera* morphotypes (Supplementary File 7). Two frameshift variants were detected within the *UFO* gene (Cp4.1LG20g02860) and were different between the two subspecies (Supplementary File 8). Among the ethylene-related genes involved in signaling, *EIN3* (Cp4.1LG04g11790) showed a frameshift mutation specific of the subspecies *ovifera* morphotypes similar to *UFO* (Supplementary File 9). This is one of the two *EIN3* genes that map in the CP32_scaffold000025. This scaffold mapped to LG3 in the map version reported by Esteras et al.^13^ where a QTL related with early flowering was located^14^. Recently, using a transcriptomic approach to study parthenocarpy in zucchini, the *EIN3* gene was found to be significantly down-regulated during fruit set, indicating that ethylene production should be limited for proper fruit set^47^. Therefore, the mutation within this gene could have paramount importance for fruit-set-related mechanisms among morphotypes. These mutations in the *UFO* and *EIN3* genes are good candidates to explain some of the flowering differences found between the two subspecies.

Other high impact mutations affected genes associated with fruit color. A frameshift mutation specific of the only yellow-fruited morphotype of the subspecies *ovifera* (the only one in the set with yellow flesh), Crookneck, was found in the Cauliflower *OR* (Orange) protein (Cp4.1LG13g00690) (Supplementary File 10). In melon, flesh color is controlled by two major genes, green flesh (*gf*) and white flesh (*wf*). A detailed mapping approach identified the Orange gene, *CmOr*, the melon homolog of the cauliflower *Or* gene^48^, as the previously described *gf locus* in melon. The function of *OR* is to induce the differentiation of plastids into chromoplasts for carotenoid accumulation. The *C. pepo* ortholog could be the one responsible for the yellow color found in this *ovifera* morphotype. It is possible that *OR* works in association with a DnaJ-like protein to bind to proteins specific for plastid differentiation/division. No high impact mutations were found in the DNAJ protein underlying the QTLs involved in flesh color (*MbFCo_19* and *MaFCo_19*, in Cp4.1LG14), Cp4.1LG14g03900 and Cp4.1LG14g03230, but some moderate variants in carotenoid cleavage dioxygenase (*CCD*) (missense_variant Ile395Val), also underlying these QTLs, are specific of the cultivar of the Scallop morphotype, the only one with white flesh and rind (Supplementary File 11).

Flesh and rind color are controlled by different genomic regions in *C. pepo*. The major QTLs controlling rind color (ILRCo_4 and IbRCo_4 MLRCo_4 and MbRCo_4) are mapped in Cp4.1LG05. We have found some mutations with moderate effect (missense variants Thr161Ser, Glu135Gly, and Ala75Pro) in the ARABIDOPSIS PSEUDO RESPONSE REGULATOR2-LIKE gene (*APRR-2-like* gene, Cp4.1LG05g02060) (Supplementary File 12) that are specific to both yellow-fruited accessions (Crookneck, homozygous, and yellow Zucchini, heterozygous), although their sequences were not found in the Scallop and Acorn morphotypes. Another mutation with moderate effect (missense variant Gln272Arg) was found in the *APRR-2-like* gene (Cp4.1LG05g02070) (Supplementary File 13) specific to the yellow Crookneck and weakly pigmented Scallop morphotypes. Genes of this family have been demonstrated to act as fruit-related regulators of pigment accumulation in tomato and pepper, so further analyses are necessary to confirm the involvement of these mutations with rind color variation in *C.pepo*.

The genetic basis of variation in fruit shape has been studied extensively as it represents an important horticultural trait associated with consumer preference, total yield, and postharvest handling-related parameters^49^. Specifically, in tomato, there are four key genes responsible for the various fruit shapes, that is, SUN and OVATE for elongated fruits, as well as LC and FAS, which encode WUSCHEL and CLAVATA3, respectively, for meristem size and locule number^50^. In *C. pepo*, a major QTL involved in fruit shape of immature and mature fruits (*IFSh_3*, *IFLe_3*, *IFWi_3*, *MFSh_3*, *MFLe_3*, and *MFWi_3*) has been reported^13,14^. Although a gene of the Ovate family (Cp4.1LG03g03420) co-localizes with this QTL, no particular mutation with high impact was detected within this gene (Supplementary File 14).

On the other hand, a SNP with high impact was detected in another gene previously reported to be involved in fruit morphology, annotated as protein IQ-DOMAIN 14-like (Cp4.1LG03g08410) (Supplementary File 15). The IQ67 domain (IQD) family includes proteins that contain the IQ67 domain, a domain that interacts with calmodulin and regulates plant growth and metabolism^49^. Known members of this family include a tomato IQD12 protein encoded by the *SUN* gene that controls fruit shape through a retrotransposon duplication event that increases the expression of this gene and imparts an elongated shape to the fruits^51^. In watermelon, a putative fruit-shape-controlling gene that bears a 159 bp deletion results in the elimination of 53 amino acids from the corresponding protein; this gene is present in an elongate-fruited watermelon cultivar and is homologous to the *Arabidopsis* IQ26 gene^52^. Similarly in cucumber, it was found that a candidate gene within the major QTL for fruit size encodes a *SUN* homolog that also has a 161 bp deletion in a round-fruited cultivar, and at the same time its expression is lower than in the long-fruited cultivar. In melon, *CmSUN14* is a cucumber *SUN* homolog and a candidate for fruit shape QTLs^53^. No particular deletions for these genes were found within our accessions. Detailed expression analyses in the flowers and fruits would indicate whether fruit-shape differences are due to variations in gene expression of the Cp4.1LG03g08410. Apart from fruit morphology and phenotype, proteins of the ovate family have recently been found to regulate fruit ripening in banana^54^, and fruit quality in tomato^55^.

Also, the YABBY transcription factor (Cp4.1LG05g04630), reported in other crops to be involved in determining fruit morphology, was located in the interval of the QTL *MFSh_4*, Mature fruit shape (Cp4.1LG05,2342850,2916621), contains SNPs with moderate predicted effects specific to subspecies *ovifera* (Supplementary File 16). In tomato, a large insertion in the first intron of a *YABBY-like* transcription factor, called *Fasciated* (*FAS*), reduces the expression of the gene and causes the high-locule number phenotype^56^ significantly affecting fruit shape. Tomato has eight more members in the *YABBY* family expressed differently in the diverse reproductive and vegetative tissues^57^, indicative of the diverse roles that these genes may play in plants. *YABBY* protein family members are essential for the establishment of the abaxial cell surface in leaves, flowers, and ovules^58^. The fact that SNPs were detected only in subspecies *ovifera* could mean that these can be used as markers for the subspecies, although further investigation is needed to establish a causal relationship with fruit shape.

**Fig. 7:**
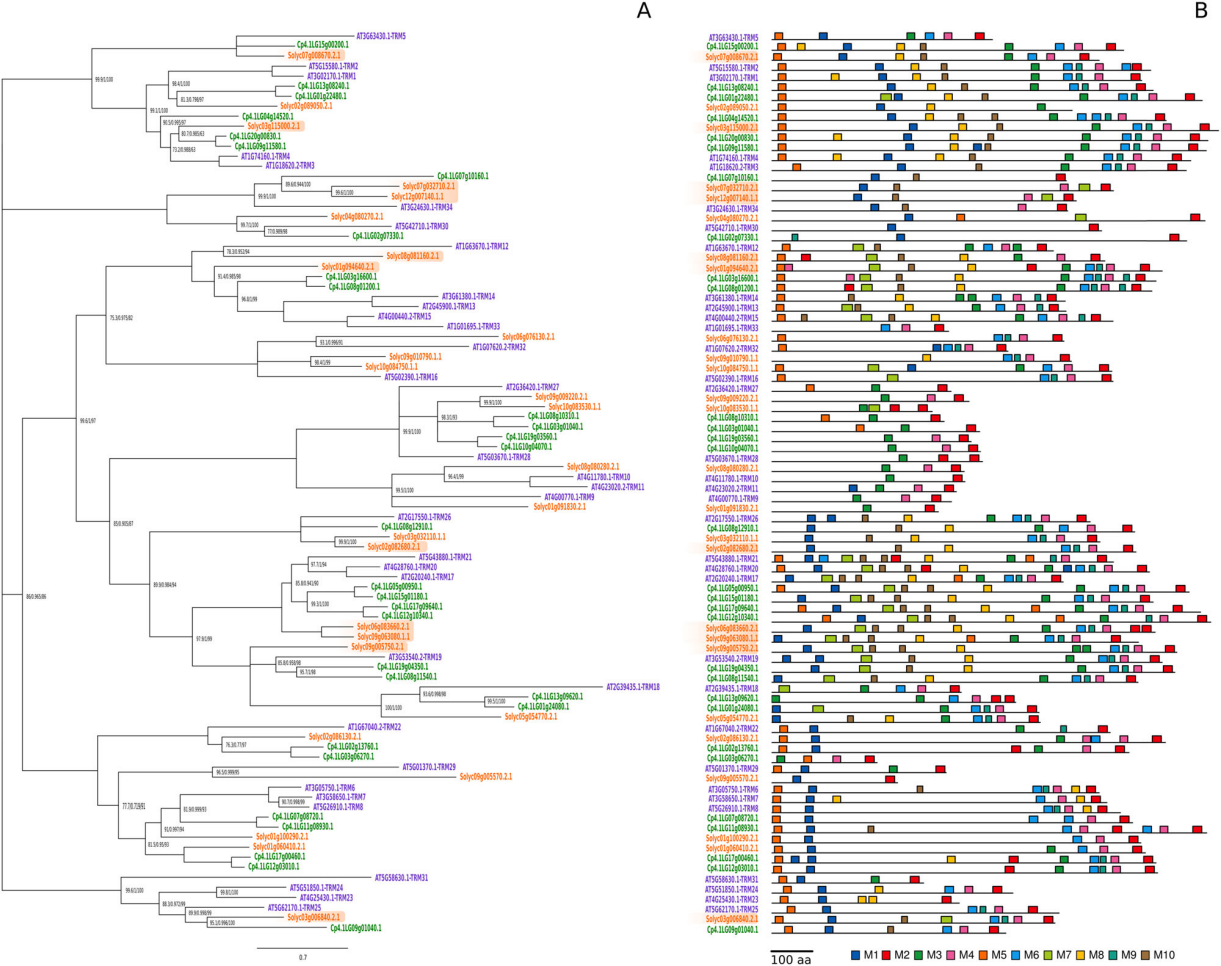
Tonneau recruiting motif structure. **a** Unrooted maximum-likelihood tree of *Cucurbita pepo* (purple), *Arabidopsis thaliana* (green), and *Solanum lycorpersicum* (red) orthologs containing a Tonneau recruiting motif. Node support expressed as Shimodaira–Hasegawa approximate likelihood -ratio (SH-aLRT) support (%)/a Bayes support/ultrafast bootstrap support (%). Nodes with a SH-aLRT lower than 70 have been collapsed with their parent node. Labels of nodes with a 100/1/100 support have been omitted for clarity. **b** Distribution of conserved motifs for each of the orthologs containing a Tonneau recruiting motif found by MEME. Sequences showing a M10 motif are likely to interact with OVATE

Recently, OVATE proteins have been reported to interact with TRM proteins to regulate cell division patterns in ovary development to alter final fruit shape^35^. Ovate proteins are known to be involved in determining fruit shape in a variety of species including tomato^59^, and pepper^60^, while in *Arabidopsis* it was shown that OFP1 targets *AtGA20ox1* repressing cell elongation^61^. A gene of the *Ovate* family co-localizes with the QTL Cp4.1LG03g03420, which has been reported as a major QTL involved in fruit shape of immature and mature fruits (IFSh_3, IFLe_3, IFWi_3, MFSh_3, MFLe_3, and MFWi_3)^13,14^. Albeit no mutations with high impact were found within the coding sequence of this gene (Supplementary File 13). Furthermore, we have identified a total of 30 putative orthologs to *Arabidopsis thaliana* and *Solanum lycopersicum* TRM proteins in *C. pepo* (Fig. 7a), some of them containing the M10 motif that is the putative motif that interacts with OVATE (named as M8 in Wu et al.^35^). Some of these genes display SNPs with high impact on the corresponding protein, located in LG3, 5, 8, 10, 12, and 15 (Cp4.1LG03g01040, Cp4.1LG03g06270, Cp4.1LG05g00950, Cp4.1LG08g10310, Cp4.1LG10g04070, Cp4.1LG12g10340, Cp4.1LG15g00200, Cp4.1LG15g01180, and Cp4.1LG17g00460) (Supplementary File 17–25). Some of them are specific to subspecies *ovifera*, such as the SNPs within Cp4.1LG03g06270, Cp4.1LG08g10310, Cp4.1LG10g04070 Cp4.1LG12g10340, Cp4.1LG15g00200, and Cp4.1LG17g00460 (Supplementary File 16–21). Others are morphotype-specific, such as those of Cp4.1LG05g00950 (Supplementary Table 22), specific of Cocozelle, the morphotype with the longest fruits, although no underlying QTLs controlling fruit shape have been previously associated with these genes. Among these genes, Cp4.1LG15g00200 is located in close phylogenetic proximity to Solyc07g008670, which is the tomato *TRM5* homolog (Fig. 7). In tomato, *TRM5* was very recently found to alter fruit shape, especially when expressed in *ovate/sov1* mutants and is suggested to regulate cell number in the proximal–distal and medial–lateral direction of the fruit^62^. In cucumber, *TRM5* is the gene underlining the fs2.1 QTL regulating fruit shape^35^. Two SNPs within Cp4.1LG15g00200 with high effect were identified in our study: one of them is producing a stop codon and is present in Cocozelle, Marrow, and Pumpkin, three morphotypes that form a unique phylogenetic clade. The function of these genes should be further elucidated in different genetic backgrounds.

## Conclusions

This is the first whole-genome analysis covering the majority of the morphotypes of *C. pepo*. Millions of SNPs were identified, and their distribution over the genome was remarkably homogeneous. As expected, using the reference genome of the subsp. *pepo* Zucchini, the accessions belonging to the subsp. *ovifera* morphotypes Acorn, Scallop, and Crookneck, presented almost twice as many SNPs than their subsp. *pepo* counterparts. Interestingly, even though both the Cocozelle and Zucchini morphotypes are quite elongate and of Italian origin, the Cocozelle accession had a greater number of SNPs than the Pumpkin and Vegetable Marrow accessions. Some morphotype-specific genes have been localized. Linkage disequilibrium was greater in subsp. *ovifera* than in subsp. *pepo*, perhaps reflective of the earlier differentiation of morphotypes within subsp. *ovifera*. Genomic regions that may have been fixed during the independent evolution and domestication of the subspecies have been identified.

The SNPs herein identified can be further deployed in genetic mapping of horticulturally important traits in segregating populations among the *C. pepo* morphotypes. Additionally, the SNPs with a high predicted effect should be further checked and validated in a larger number of accessions from each of the morphotypes, to confirm and expand our knowledge of allelic effects, and aid efforts to reveal the specific molecular mechanisms controlling the expression of horticulturally valuable traits.

## Supplementary information

Additional Information

Supplementary Table 1.

Supplementary Table 2.

Supplementary Table 3.

Supplementary Table 4.

Supplementary Table 5.

Supplementary Table 6.

Supplementary Table 7.

Supplementary Table 8.

Supplementary Table 9.

Supplementary Table 10.

Supplementary Table 11.

Supplementary Table 12.

Supplementary Table 13.

Supplementary Table 14.

Supplementary Table 15.

Supplementary Table 16.

Supplementary Table 17.

Supplementary Table 18.

Supplementary Table 19.

Supplementary Table 20.

Supplementary Table 21.

Supplementary Table 22.

Supplementary Table 23.

Supplementary Table 24.

Supplementary Table 25.

## Data Availability

All of the raw reads of the rapeseed accessions generated in this study have been deposited in the public database of National Center of Biotechnology Information under PRJNA523120 (Release date: 31-03-2019).
