# Supplementary material for: Whole-genome resequencing of Cucurbita pepo morphotypes to discover genomic variants associated with morphology and horticulturally valuable traits
Source: Hortic Res. 2019 Aug 11;6:94. doi: 10.1038/s41438-019-0176-9 (PMC6804688; doi:10.1038/s41438-019-0176-9)
Supplement: Supplementary file 1 — Additional Information [file 41438_2019_176_MOESM1_ESM.docx]

**Whole-genome resequencing of *Cucurbita pepo* morphotypes to discover genomic variants associated with morphology and horticulturally valuable traits**

Aliki Xanthopoulou‡, Javier Montero-Pau‡, Ifigeneia Mellidou, Christos Kissoudis, José Blanca, Belén Picó, Aphrodite Tsaballa, Eleni Tsaliki, Athanasios Dalakouras, Harry S. Paris, Maria Ganopoulou, Theodoros Moysiadis, Maslin Osathanunkul, Athanasios Tsaftaris, Panagiotis Madesis^*^, Apostolos Kalivas, Ioannis Ganopoulos^*^

**Supplementary data**

Supplementary Files 1-25

**Supplementary Files**

Supplementary File 1. List of unique genes per morphotype with SNPs with a high or moderate predicted effect.

Supplementary File 2. List of genes of *Cucurbita pepo* subsp. *pepo* with a genetic diversity higher than 99% of the genetic diversity distribution for all the genes of the subspecies. High genetic diversity genes that are shared with subsp. *ovifera* have been omitted. GeneID, predicted function, Fst between the two subspecies, genetic diversity and Tajima's D are shown for each gene.

Supplementary File 3. List of genes of *Cucurbita pepo* subsp. *ovifera* with a genetic diversity higher than 99% of the genetic diversity distribution for all the genes of the subspecies. High genetic diversity genes that are shared with subsp. *pepo* have been omitted. GeneID, predicted function, Fst between the two subspecies, genetic diversity and Tajima's D are shown for each gene.

Supplementary File 4. List of genes with Fst = 1.00 distributed in all chromosomes.

Supplementary File 5. Genes of horticultural interest. Selected genes, genomic locations, traits affected, number of changes predicted in total and with high and moderate effect, and number of changes 5 Kb upstream or downstream of the genes that can have a potential effect are shown. For the TON1 recruiting motif proteins, asterisks identify those proteins that are phylogenetically close to TRM proteins of tomato known to interact with OVATE.

Supplementary File 6. List of SNPs within the Flowering time control protein (*FPA*) gene (Cp4.1LG17g10910) with a high or moderate predicted effect that represent a private allele in a given morphotype.

Supplementary File 7. List of SNPs within the Squamosa promoter binding protein-like gene (Cp4.1LG17g10620) with a high or moderate predicted effect that represent a private allele in a given morphotype.

Supplementary File 8. List of SNPs within the Unusual Floral Organs (*UFO*) gene (Cp4.1LG20g02860) with a high or moderate predicted effect that represent a private allele in a given morphotype.

Supplementary File 9. List of SNPs within the Ethylene Insensitive 3 (*EIN3*) gene (Cp4.1LG04g11790) with a high or moderate predicted effect that represent a private allele in a given morphotype.

Supplementary File 10. List of SNPs within the Cauliflower Orange gene (Cp4.1LG13g00690) with a high or moderate predicted effect that represent a private allele in a given morphotype.

Supplementary File 11. List of SNPs within the Carotenoid cleavage dioxygenase (*CCD*) gene (Cp4.1LG14g02990) with a high or moderate predicted effect that represent a private allele in a given morphotype.

Supplementary File 12. List of SNPs within the ARABIDOPSIS PSEUDO RESPONSE REGULATOR2-LIKE (*APRR2-1* Cp4.1LG05g02060 and *APRR2-2* Cp4.1LG05g02070) gene with a high or moderate predicted effect that represent a private allele in a given morphotype.

Supplementary File 13. List of SNPs within the ARABIDOPSIS PSEUDO RESPONSE REGULATOR2-LIKE (*APRR2-1* Cp4.1LG05g02060 and *APRR2-2* Cp4.1LG05g02070) gene with a high or moderate predicted effect that represent a private allele in a given morphotype.

Supplementary File 14. List of SNPs within the OVATE gene (Cp4.1LG03g03420) with a high or moderate predicted effect that represent a private allele in a given morphotype.

Supplementary File 15. List of SNPs within the IQ-DOMAIN 14-like gene (Cp4.1LG03g08410), with a high or moderate predicted effect that represent a private allele in a given morphotype.

Supplementary File 16. List of SNPs within the YABBY transcription factor (Cp4.1LG05g04630), with a high or moderate predicted effect that represent a private allele in a given morphotype

Supplementary Files 17. List of SNPs within TONNEAU1 Recruiting Motif proteins, with a high or moderate predicted effect that represent a private allele in a given morphotype

Supplementary File 18. List of SNPs within TONNEAU1 Recruiting Motif proteins, with a high or moderate predicted effect that represent a private allele in a given morphotype

Supplementary File 19. List of SNPs within TONNEAU1 Recruiting Motif proteins, with a high or moderate predicted effect that represent a private allele in a given morphotype

Supplementary File 20. List of SNPs within TONNEAU1 Recruiting Motif proteins, with a high or moderate predicted effect that represent a private allele in a given morphotype

Supplementary File 21. List of SNPs within TONNEAU1 Recruiting Motif proteins, with a high or moderate predicted effect that represent a private allele in a given morphotype

Supplementary File 22. List of SNPs within TONNEAU1 Recruiting Motif proteins, with a high or moderate predicted effect that represent a private allele in a given morphotype

Supplementary File 23. List of SNPs within TONNEAU1 Recruiting Motif proteins, with a high or moderate predicted effect that represent a private allele in a given morphotype

Supplementary File 24. List of SNPs within TONNEAU1 Recruiting Motif proteins, with a high or moderate predicted effect that represent a private allele in a given morphotype

Supplementary File 25. List of SNPs within TONNEAU1 Recruiting Motif proteins, with a high or moderate predicted effect that represent a private allele in a given morphotype
